# Reversible Electrochemical Energy Storage Based on Zinc-Halide Chemistry

**DOI:** 10.1021/acsami.0c20622

**Published:** 2021-03-16

**Authors:** Andinet Ejigu, Lewis W. Le Fevre, Robert A. W. Dryfe

**Affiliations:** † Department of Chemistry, University of Manchester, Oxford Road, Manchester M13 9PL, U.K.; ‡ National Graphene Institute, University of Manchester, Oxford Road, Manchester M13 9PL, U.K.; § Department of Electrical and Electronic Engineering, University of Manchester, Oxford Road, Manchester M13 9PL, U.K.; ∥ Henry Royce Institute, University of Manchester, Oxford Road, Manchester M13 9PL, U.K.

**Keywords:** Zn-ion battery, halogen conversion intercalation, halogen conversion adsorption, halogen cathode, carbon−Zn halide composite, Zn-ion capacitor

## Abstract

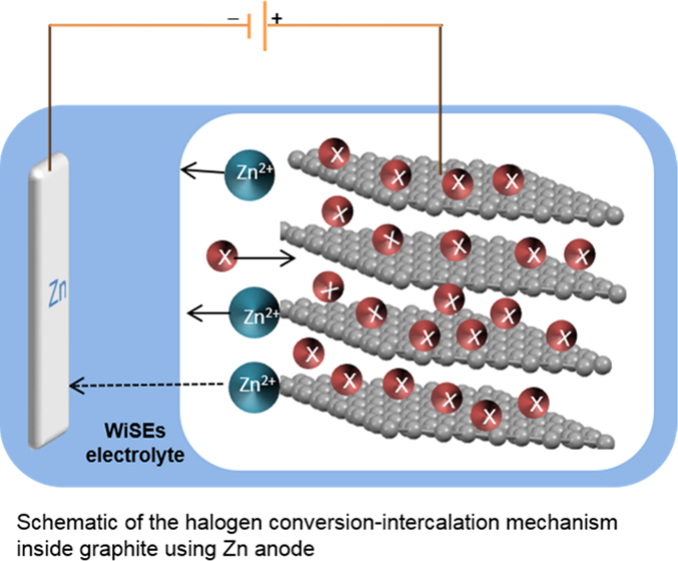

The development of rechargeable Zinc-ion batteries (ZIBs) has been hindered by the lack of efficient cathode materials due to the strong binding of divalent zinc ions with the host lattice. Herein, we report a strategy that eliminates the participation of Zn^2+^ within the cathode chemistry. The approach involves the use of composite cathode materials that contain Zn halides (ZnCl_2_, ZnBr_2_, and ZnI_2_) and carbon (graphite or activated carbon), where the halide ions act both as charge carriers and redox centers while using a Zn^2+^-conducting water-in-salt gel electrolyte. The use of graphite in the composite electrode produced batterylike behavior, where the voltage plateau was related to the standard potential of the halogen species. When activated carbon was used in the composite, however, the cell acted as a hybrid Zn-ion capacitor due to the fast, reversible halide ion electrosorption/desorption in the carbon pores. The ZnX_2_-activated carbon composite delivers a capacity of over 400 mAh g^–1^ and cell energy density of 140 Wh kg^–1^ while retaining over 95% of its capacity after 500 cycles. The halogen reaction mechanism has been elucidated using combinations of electrochemical and *in situ* spectroscopic techniques.

## 1 Introduction

Aqueous rechargeable batteries are a promising class of batteries for grid-scale electrochemical energy storage owing to their low cost, ease of fabrication, high ionic conductivity, and high operational safety.^1−3^ Research on aqueous batteries in recent years has been gaining momentum from application in low-voltage divalent zinc–ion batteries (ZIB) to high-voltage monovalent lithium-ion batteries (LIBs).^4−6^ In particular, ZIBs have attracted substantial interest as one of the most promising next-generation technologies because: (i) they depend on an Earth-abundant metal, which is air-stable unlike Li; (ii) their low cost, safety, and environmental benignancy is attractive for grid-scale energy storage, and (iii) the volumetric energy density is approximately 3 times higher than that of Li.^2,7,8^ Due to these favorable properties, zinc has been used as an anode material in a series of battery technologies both in conventional static cells (zinc–manganese dioxide batteries, zinc–air batteries, or Zn-graphite dual ion batteries) and in redox flow configurations (Zn–bromine or Zn–iron cells).^9,10^

The development of ZIBs is, however, hindered by a number of factors relating to aqueous electrolytes, the formation of Zn dendrites at the anode, and lack of efficient cathode materials.^7^ Furthermore, the codecomposition of water molecules during the deposition of Zn^2+^ is known to affect the reversibility of the Zn stripping/deposition and depletes the electrolyte due to the sustained water consumption. Wang et al. used water-in-salt electrolytes (WiSEs) to enhance the electrochemical window of water and obtained dendrite-free Zn plating/stripping with near 100% Coulombic efficiency.^7^ WiSEs contain a high concentration of the desired salt so that the hydrated ions outnumber free water: as there is no free water to react at the electrode surface, the overall cell voltage can be increased. The combination of small cations and large fluorinated anions in water alters the hydration behavior of the ions where the cation is strongly solvated, but the anion is not. The less solvated fluorinated anions can be reduced to form a passivating solid electrolyte interface (SEI) on the electrode surface.^5^ This SEI formation significantly suppresses the hydrogen evolution reaction and is largely responsible for the overall electrochemical stability window of WiSEs.^5^ The highest voltage window (4.9 V) recorded at the hydrophobic graphite of WiSEs contains small metal cations (Li^+^) and large fluorinated anions such as bis(trifluoromethanesulfonyl)imide ([TFSI]^−^) and trifluoromethanesulfonate ([TFO]^−^).^6^

Although the electrochemical reversibility of Zn stripping/deposition was enhanced and Zn dendrite formation was suppressed using WiSEs, the lack of an efficient cathode material for ZIBs remains a severe challenge.^11,12^ Some of the cathode materials developed to date are Prussian blue analogs,^13,14^ manganese oxides,^15−18^ and vanadium oxides.^11,12^ Each of these materials suffers from limited specific capacity, far below the theoretical capacity of the Zn anode (820 mAh g^–1^), a low-voltage plateau (<1.4 V), and a low rate capability due to their poor electronic conductivity. The low performance of these cathodes is mainly attributed to the high polarization of the divalent Zn^2+^ ions leading to strong binding with the host lattice and sluggish solid-state migration dynamics.^1,12^ Therefore, the development of efficient cathodes should use different combinations of battery chemistry, for example, reversible deposition and stripping of Zn as the anode half-reaction and reversible atomic intercalation or plating of non-Zn-ion species as the cathode half-reaction. This strategy not only avoids the intercalation of Zn^2+^ to the host cathode material but also allows the flexibility to fine tune and increase the output voltage, which is the main barrier limiting the overall performance.

Herein, we describe a new class of composite cathode materials that contain Zn halides (ZnCl_2_, ZnBr_2_, and ZnI_2_) and carbons (graphite or activated carbon), where the halide ions act both as charge carriers and as redox centers. It should be noted that our approach differs from the conventional Zn–bromine flow battery, where bromide is oxidized to bromine and stored in an external tank.^19^ The approach we pursue in the current study uses Zn halide immobilized on carbon hosts as the cathode, where the redox activity of the halides is exploited to store more charge. In other words, the halides undergo a “conversion–intercalation/adsorption” reaction inside the carbon structure when the cell is charged using a Zn^2+^ conducting water-in-trisalt (WiTS) gel electrolyte. Our approach also removes the halogen cross-over seen in Zn–bromine cells as the halogen is confined (intercalated or adsorbed) within the carbon structure upon oxidation. The variety of the halogen species means that the cell voltage can be fine-tuned and increased. The standard reduction potentials of I^–^/I_2_, Br^–^/Br_2_, Cl^–^/Cl_2_, and F^–^/F_2_ redox couples are 0.54, 1.09, 1.36, and 2.8 V vs standard hydrogen electrode (SHE), respectively.^20^ The combination of the zinc-halide–carbon cathode with a Zn anode can therefore generate an open-circuit voltage that ranges between 1.3 and 3.5 V depending on the halide type. The flexible and non-flammable semisolid WiTS gel electrolyte, which is compatible with the halogen cathode, exhibited fast electrode kinetics for Zn oxidation and reduction without the formation of Zn dendrites. We will show that both the identity of the Zn halide and carbon structure in the cathode produces electrochemical energy storage devices that fundamentally differ from one another. The resultant composite electrodes can deliver a capacity of 480 mAh g^–1^ at 0.05 A g^–1^ with a corresponding energy density of 140 Wh kg^–1^. The conversion–intercalation/adsorption mechanism of the individual ZnX_2_–carbon composite in the WiTS gel electrolyte is characterized by electrochemical, *in situ* Raman spectroscopy and *ex situ* X-ray photoelectron spectroscopy (XPS) techniques and analyzed in detail.

## 2 Results and Discussion

### 2.1 WiTS Gel Electrolyte Formulation and Characterization

**Figure 1:**
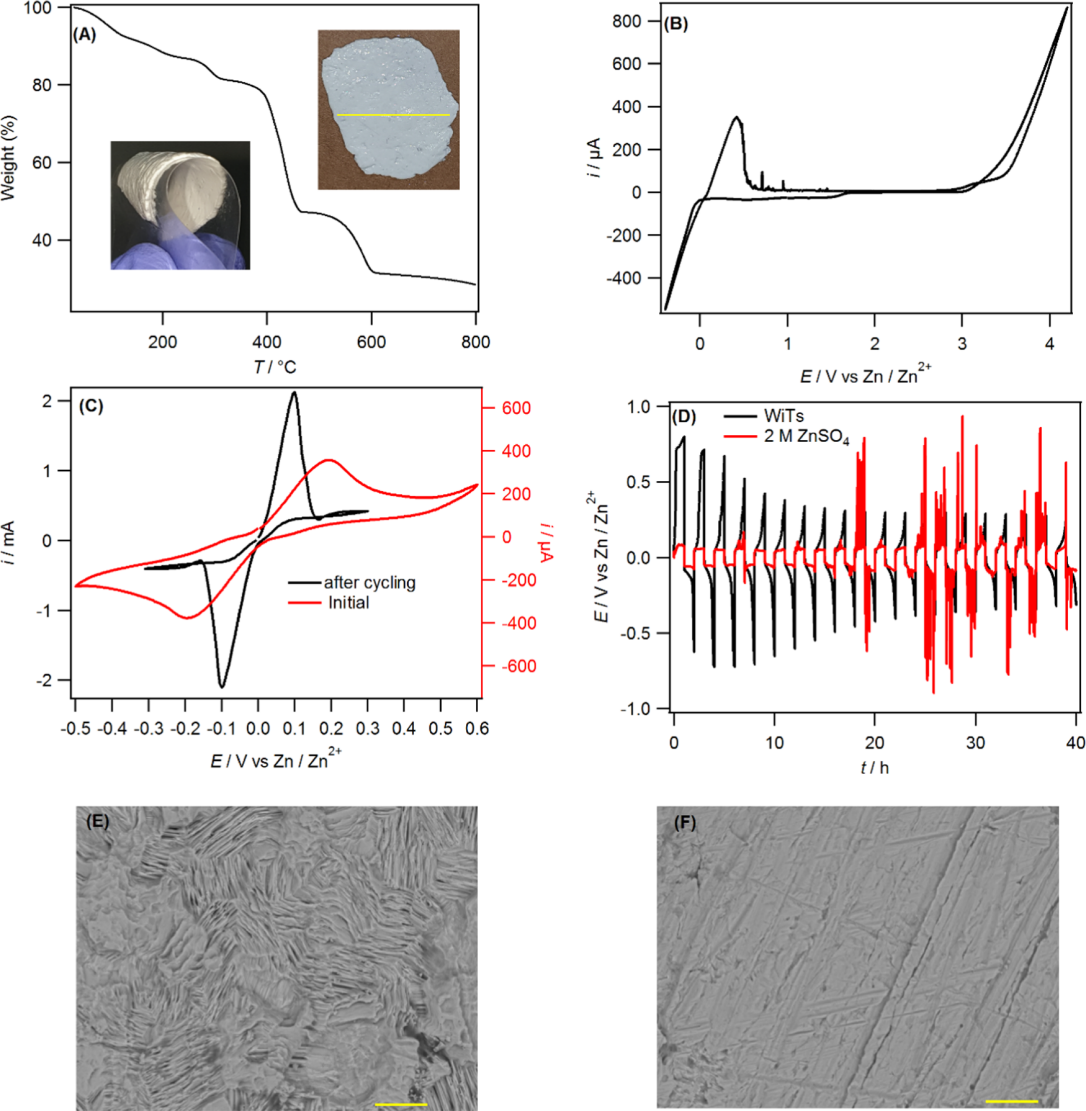
(A) Thermogravimetric analysis (TGA) traces recorded for a WiTS gel electrolyte obtained by ramping the temperature from 30 to 800 °C at a rate of 10 °C min^–1^ under N_2_. The inset shows the photographic images of a WiTS gel electrolyte, the scale bar is 5 cm. (B) Cyclic voltammetry (CV) recorded at a 3 mm diameter glassy carbon (GC) electrode at 10 mV s^–1^ using a WiTS gel electrolyte between −0.2 and 4.2 V. (C) CVs recorded with a symmetrical Zn|Zn cell at 1 mV s^–1^ in a WiTS gel electrolyte before and after cycling for 40 h using galvanostatic charge–discharge. (D) Galvanostatic charge–discharge curve obtained using a symmetrical Zn|Zn cell in a WiTS gel electrolyte and 2.0 M ZnSO_4_ (aq) at 0.2 mA cm^–2^. (E) Scanning electron microscopy (SEM) image of a fully discharged Zn substrate, the scale bar = 20 μm. (F) SEM of a fully charged Zn substrate, scale bar = 20 μm.

The robust Zn^2+^ conducting gel electrolyte was formulated from water-in-trisalt (WiTS) electrolyte by mixing ZnSO_4_, Zn(TFO)^2^, and LiTFSI (in a 1:1:2 mass ratio, respectively) in 20% water using 10% poly(tetrafluoroethylene) (PTFE) polymer binder. The resulting white gel is a highly flexible, yet semisolid, electrolyte that can be molded to any shape (inset of Figure 1A for optical image).

Each of the components contributes to the unique properties of the gel. LiTFSI was used as it is soluble in water to a high concentration (when compared to Zn(TFO)^2^ or ZnSO_4_) to reach the water-in-salt regime. It is also used as a source of the [TFSI] anion since it is believed that the reduction of [TFSI]^−^ is responsible for the formation of the passivating SEI, which extends the overall electrochemical window.^5,7^ We also note that in the absence of ZnSO_4_ the gel is very sticky to handle, while in the absence of Zn(TFO)^2^, a rigid dry material is formed, as excess ZnSO_4_ pulls water from the mixture. The thermal decomposition of the gel electrolyte was studied by thermogravimetric analysis (TGA), which shows four mass losses due to the loss of H_2_O (<200 °C), decomposition of [SO_4_]^2–^ (200–284 °C, ∼10%), [TFO]^−^ (323–450 °C, ∼34%), and [TFSI]^−^ (>450 °C).^21−23^ The TGA also shows the gel contained about 15% water. The ionic conductivity of the gel electrolyte was determined using alternating current (AC) impedance (Figure S1) and was found to be 6 mS cm^–1^, which is comparable to that of nonaqueous electrolytes (9.0 mS cm^–1^) used in commercial LIBs.^5^ The electrochemical window of the gel, as well as the reversibility of Zn plating and stripping, was investigated using cyclic voltammetry (CV) at a glassy carbon (GC) disk electrode. The gel electrolyte exhibited fast kinetics for Zn oxidation/reduction and achieved a potential window of 3.0 V (Figure 1B), which is comparable to WiSE-based on 21 m LiTFSI.^5^

The long-term electrochemical reversibility of the Zn plating and stripping processes in the WiTS gel electrolyte was investigated using a Zn|Zn symmetric cell under galvanostatic and CV methods. Figure 1C shows the CV obtained before and after several charge–discharge cycles; and in each case, the gel electrolyte exhibited reversible Zn redox chemistry with the ratio between the anodic (*i*_p,a_) and cathodic peak currents (*i*_p,c_) being one which indicates that the Coulombic efficiency of the cell is near 100%. The kinetics of Zn oxidation/reduction, however, significantly improved after cycling, as exemplified by the peak-to-peak (Δ*E*_P_) separation that decreased from ∼0.4 to 0.2 V. The decrease in Δ*E*_P_ with cycling is most likely due to the removal of surface oxides from Zn, which impede electron transfer. In addition, the current due to Zn oxidation/reduction is increased by a factor of 5 after cycling, which could be due to an increase in the active surface area. The charge–discharge curve (Figure 1D) also showed similar behavior where the overpotential (η) for Zn stripping/plating decreased from about 0.6 to 0.2 V after 10 cycles and stabilized at 0.2 V even after the cell was cycled for 40 h at 0.2 mA cm^–2^. The cell can operate continuously without short circuiting (no formation of dendrites) for over 400 h at 0.1 mA cm^–2^ with a much lower η for Zn|Zn^2+^ redox reactions (see Figure S2). In sharp contrast, in a dilute electrolyte (2 M ZnSO_4_), a rapid polarization started to occur after nine cycles most likely due to the formation of Zn dendrites and other associated problems including oxide formation (Figure 1D).^24^ These observations demonstrate that the gel electrolyte is an excellent Zn^2+^ conductor with very facile Zn stripping/plating kinetics. Both the stability and the kinetics of the Zn couple in the WiTS gel electrolyte are superior to those in solution-based aqueous and nonaqueous ionic liquid-based electrolytes.^25−28^

The presence or absence of Zn dendrite formation in this gel electrolyte is also examined using SEM after galvanostatic cycling of a Zn|Zn symmetric cell for 40 h. The Zn-plated substrate exhibited a dense and uniform layeredlike structure with the absence of any substantial dendrites (Figure 1E). After Zn stripping, this dense structure is completely removed and the original surface is retained without the formation of ZnO according to X-ray diffraction (XRD) (Figure S3), indicating the reversibility of Zn chemistry in the gel electrolyte.

In summary, this Zn^2+^-conducting WiTS gel electrolyte provides the advantage of safety (as it is nonflammable) and enables certain battery components to be removed (such as the polymer separator). The elimination of the separator from the cell will significantly reduce the contact resistance of the interface. Then, we consider the cathode chemistry involving the carbon host.

### 2.2 Aqueous Zn|(Graphite–ZnX_2_) Cell

**Figure 2:**
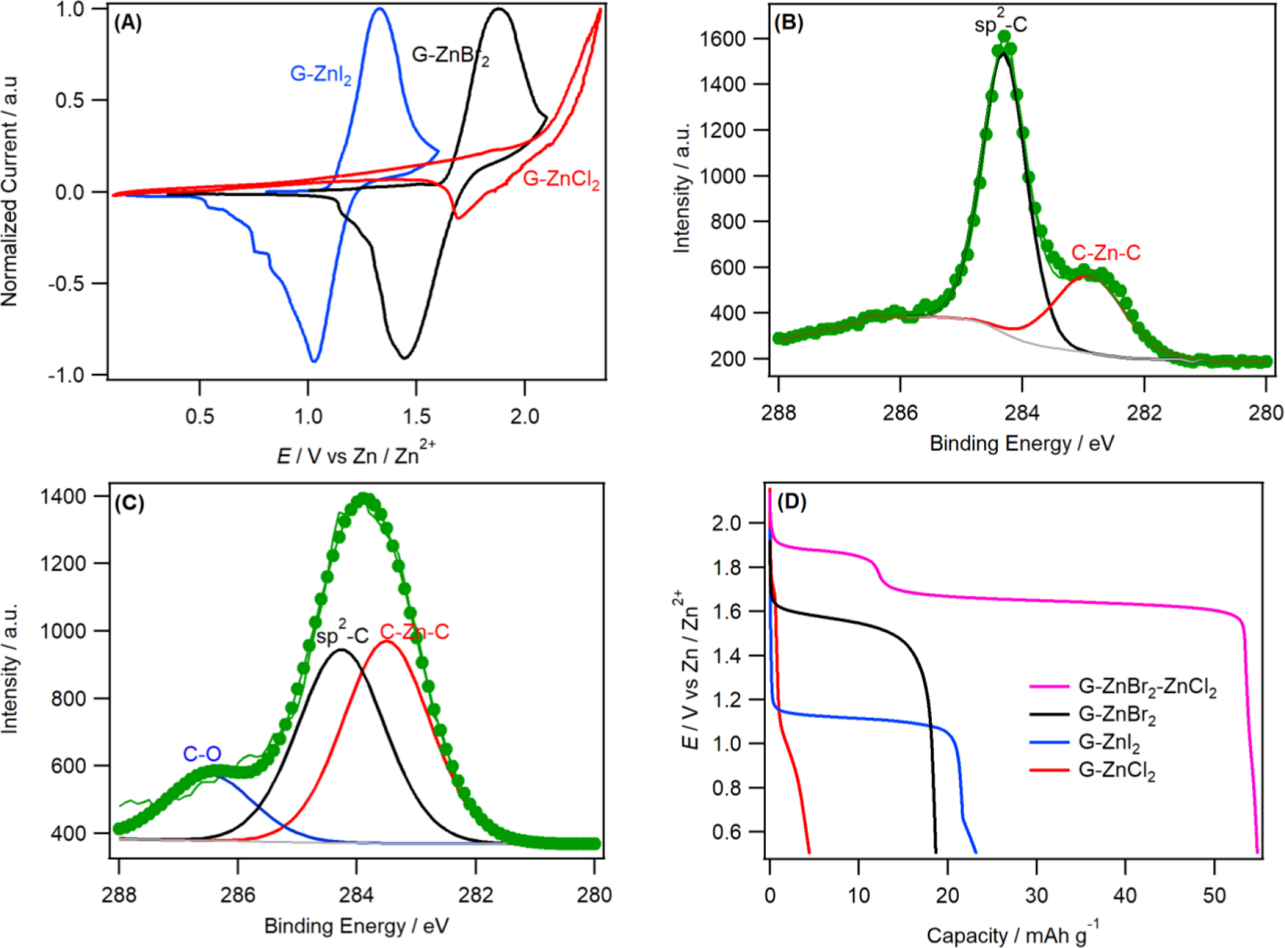
(A) Cyclic voltammograms recorded at 1 mV s^–1^ in the WiTS gel electrolyte using coin cells constructed from G–ZnX_2_ positive electrodes and a Zn foil negative electrode. *Ex situ* XPS of G–ZnCl_2_–ZnBr_2_ obtained for (B) fully charged and (C) fully discharged cells. (D) Galvanostatic discharge curves vs capacity obtained at a current density of 50 mA g^–1^ using the positive electrodes (G–ZnX_2_) shown and the Zn negative electrode in the WiTS gel electrolyte.

The electrochemistry of confined Zn halides within a graphite electrode was examined using the WiTS gel electrolyte in full-cell Zn batteries. The free-standing graphite–ZnX_2_ cathodes were prepared by mixing the desired halide and natural graphite at a mass ratio of 1:3 with 5% PTFE binder. Figure 2A shows the CVs recorded at the cathodes of graphite–ZnI_2_ (G–ZnI_2_), graphite–ZnBr_2_ (G–ZnBr_2_), and graphite–ZnCl_2_ (G–ZnCl_2_) combined with Zn anodes in cells using the WiTS gel electrolyte. Significantly, both G–ZnI_2_ and G–ZnBr_2_ showed reversible redox reactions at the characteristic formal potentials of I^–^/I_2_ (1.17 V vs Zn/Zn^2+^) and Br^–^/Br_2_ (1.67 V vs Zn/Zn^2+^). The oxidative redox reactions are therefore attributed to the conversion of the halide ion (I^–^, Br^–^) to elemental halogen (I^0^ or Br^0^), which is stabilized by sequential intercalation/adsorption into graphite galleries to form a solid graphite intercalation compound (GIC) (eq 1).^6,29−31^ This oxidation process releases Zn^2+^, which is transported through the gel electrolyte to replace the ions reversibly plated on the Zn anode (eq 2). The reduction process at the cathode is due to the deintercalation and reduction of I^0^/Br^0^ to recombine with Zn^2+^ (*n* is the molar ratio of carbon atoms to the intercalated/adsorbed halogens in the GIC). The ratio between the *i*_p,a_ and the *i*_p,c_ being one in Figure 2A demonstrates the high reversibility of eq 1
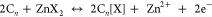
1
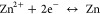
2

In contrast, the CV obtained using the G–ZnCl_2_ cathode showed a lower current and a lower degree of reversibility with a sharp undefined oxidation peak and a small reduction peak. The poor reversibility of G–ZnCl_2_ suggests the formation of an irreversible product during battery charging. This is in contrast to G–LiCl which has been reported to display a reversible Cl^–^ conversion–intercalation process in WiSEs.^6^ The fact that the conversion–intercalation of ZnCl_2_ is irreversible suggests that the metallic counterions have a strong impact on the conversion–intercalation process. It has been shown that the chemical intercalation of ZnCl_2_ into graphite forms a strong complex with the carbon species to form ZnCl_2_–carbon.^32^ Indeed, characterization of a fully charged G–ZnCl_2_-containing electrode using XPS showed the formation of C–Zn–C bonds at a low binding energy (283.0 eV) when analyzing the high-resolution C1s spectrum (Figure 2B).^33^ Furthermore, the signal due to Zn carbide is still present and increased for the fully discharged cathode, which confirms the irreversibility of the process, manifested in the CV response (Figure 2C). The slight shift to high binding energy (283.5 eV) during discharge indicates a change in the environment of C1s presumably due to the increase in the concentration of chloride along with Zn inside the carbon. This data demonstrates that ZnCl_2_-based cathode materials cannot be combined with sp^2^-carbon to form a secondary ZIB.

Figure 2D presents the discharge curves at 50 mA g^–1^of the ZIB full cells with the WiTS gel electrolyte for different halide-based cathodes. As expected, G–ZnCl_2_ showed a very low capacity with the absence of any useable voltage plateau due to the irreversible reaction. The G–ZnI_2_ and G–ZnBr_2_ cathodes showed voltage plateaus that correspond to their respective redox reactions, G–ZnI_2_ at 1.17 V and G–ZnBr_2_ at 1.67 V vs Zn/Zn^2+^, in agreement with the CV data. The cathode made from the equimolar mixture of ZnCl_2_ and ZnBr_2_ showed two discharges voltage plateaus: a small one at 1.90 V, due to the Cl^–^/Cl_2_ redox reaction, and the other at 1.67 V due to the Br^–^/Br_2_ redox reaction. Nonetheless, the specific capacity of each cathode is much lower than the theoretical capacity of a halogen GIC (309 mAh g^–1^ for MBr_n_ and 632 mAh g^–1^ for MCl_n_).^6^ Among the cathodes tested, the best specific capacity (55 mAh g^–1^) was obtained using G–ZnCl_2_–ZnBr_2_ with the others being lower than 30 mAh g^–1^. However, the capacity decayed by more than 50% after 200 cycles due to the continual formation of zinc carbide species (Figure S4). The specific capacity quoted is based on the total mass of the cathode (mass of graphite plus mass of halide).

**Figure 3:**
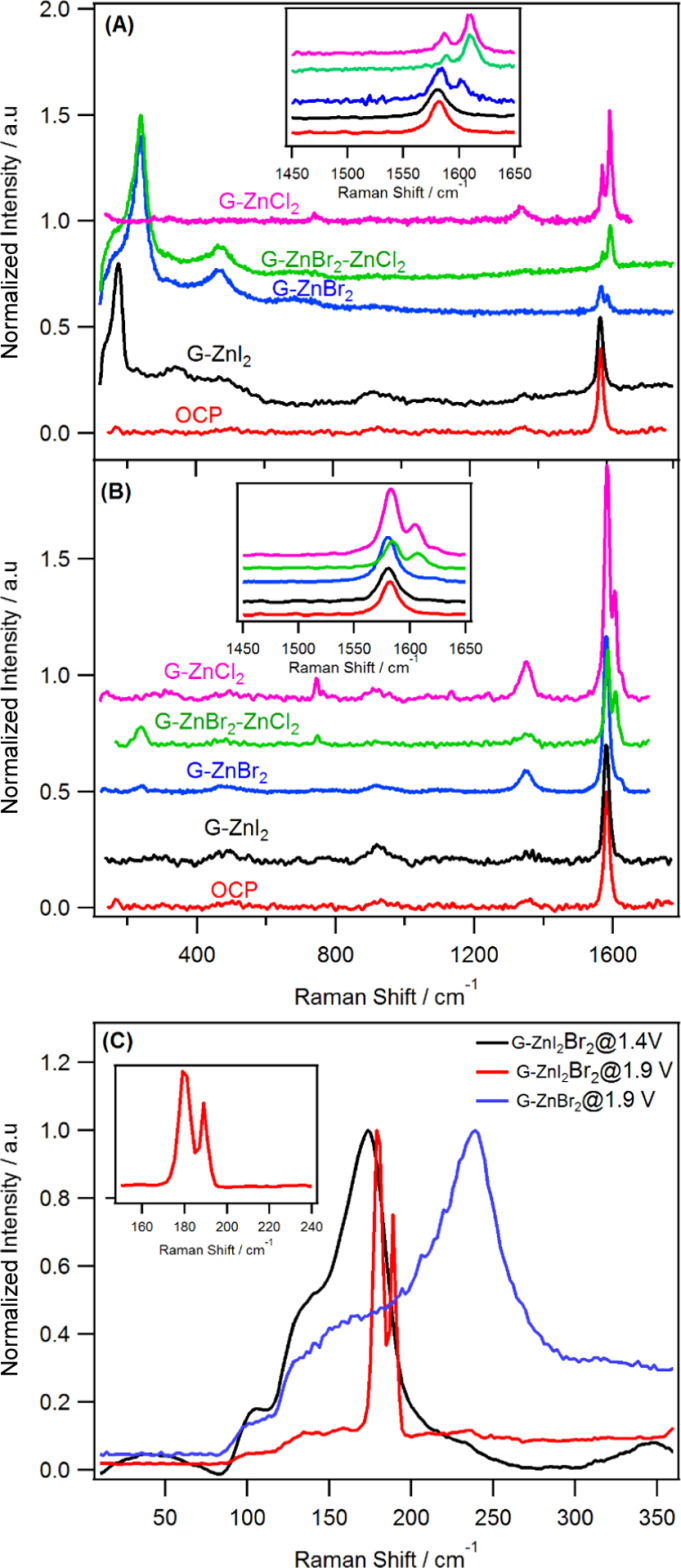
*In situ* Raman spectral series of a Zn|G–ZnX_2_ cell in the WiTS gel electrolyte during full (A) charge and (B) discharge. The insets in (A) and (B) show the graphite G-band region. (C) Comparison of Zn|G–ZnX_2_ at different voltages.

*In situ* Raman spectroscopy was used to understand the halide intercalation mechanism and to rationalize the poor performance of the G–ZnX_2_ using the WiTS gel electrolyte. Figure 3 shows the fully charged–discharged Raman spectra for each cathode. The free-standing sample for each cathode showed a similar response at open-circuit potential (OCP), with the characteristic graphite bands, G-band at 1580 cm^–1^ and a small D-band at ∼1350 cm^–1^, being shown and no other bands associated with Zn halides. The fully charged G–ZnI_2_ electrode displayed an intense Raman signal at 172 cm^–1^ due to surface-bound iodine species.^34,35^ However, the absence of a G-band splitting suggests that there is no intercalation of the iodide species into the graphite galleries (see the inset of Figure 3A). While other halogens including chlorine and bromine intercalate into graphite, iodine has a strong affinity for adsorption rather than intercalation.^36,37^ G–ZnBr_2_ also exhibited similar behavior when fully charged where it showed a signal at 240 cm^–1^ due to the stretching mode of Br_2_.^6,38^ The G-band of the material, however, was split into two Raman modes: the E_2g2i_ mode at ∼1580 cm^–1^ due to the interior unintercalated original layers and the E_2g2b_ at 1604 cm^–1^ due to the bounding layers next to the intercalants. The fact that the intensity of the E_2g2i_ mode is twice that of the E_2g2b_ together with the higher wavenumber for the E_2g2b_ reflects the dilute staging of bromine species into graphite galleries.^39^ The opposite trends were observed during discharge that involves the desorption/deintercalation of the halogen at each cathode (Figure 3B) characterized by the absence of halogen-related bands, further confirming the reversibility of the process. This *in situ* Raman spectroscopy data shows that reversible surface adsorption/desorption is the dominant reaction mechanism in G–ZnI_2_ and G–ZnBr_2_ electrodes. This observation is similar to Na chemistry at a graphite electrode where Na plates are on the graphite surface rather than intercalating due to its size and weaker chemical interaction with the graphite planes.^40^ The use of hard carbon in the composite may improve the cell performance.

The electrode that contained ZnCl_2_ (neat or mixed with ZnBr_2_) on the other hand showed a more intense E_2g2b_ at 1609 cm^–1^, approximately twice that of the E_2g2i_, a characteristic of the formation of a stage-3 GIC.^39^ However, the G-band splitting remained after the battery was fully discharged, which demonstrates that the intercalation process is nonreversible, in agreement with the XPS analysis (inset of Figure 3B). Furthermore, the fully charged G–ZnCl_2_ or G–ZnBr_2_–ZnCl_2_ did not show the band associated with Cl_2_ (expected in the region of 530–570 cm^–1^) intercalant due to the reaction of ZnCl_2_ with the graphite host as previously discussed. Figure 3C shows the Raman spectrum response for the G–ZnI_2_–ZnBr_2_ composite electrode. It is interesting to note that significant Raman frequency shifts for both iodide and Br-species were observed when analyzing the G–ZnI_2_–ZnBr_2_ sample. When the cell was charged to 1.4 V, a broad band at 172 cm^–1^ due to surface-bound iodine species (also the case for G–ZnI_2_ charged to 1.9 V) was seen. When the voltage was increased to 1.9 V, sharp bands at 180 and 189 cm^–1^ were observed along with the G-band splitting. The formation of these new bands is most likely due to the formation of interatomic IBr intercalants.^41^ A frequency downshift is often observed for surface-bound halogen when compared to free halogen due to the interaction of halogen with host materials, which weakens the interatomic bonds of the intercalants.^6,41^ In contrast, the G–ZnCl_2_–ZnBr_2_ sample did not show the BrCl formation due to the reaction of ZnCl_2_ with the graphite (Figure 3A).

Overall, the analysis of Raman spectra indicates that the size of halogens significantly impacts the reaction mechanism at the graphite cathode. The conversion–adsorption process occurs when the halogen is larger, for example in G–ZnI_2_, and the conversion–intercalation process occurs for smaller halogens such as G–ZnCl_2_. Even though ZnCl_2_ can reach a reasonably high intercalation staging, the irreversible reaction is the hindering factor for use in practical ZIBs. The low capacity of the full-cell battery at each electrode is explained by the conversion–adsorption reaction mechanism, which needs a high surface area carbon rather than the low surface area graphite.

### 2.3 Aqueous Zn|(Activated Carbon–ZnX_2_) Cell

**Figure 4:**
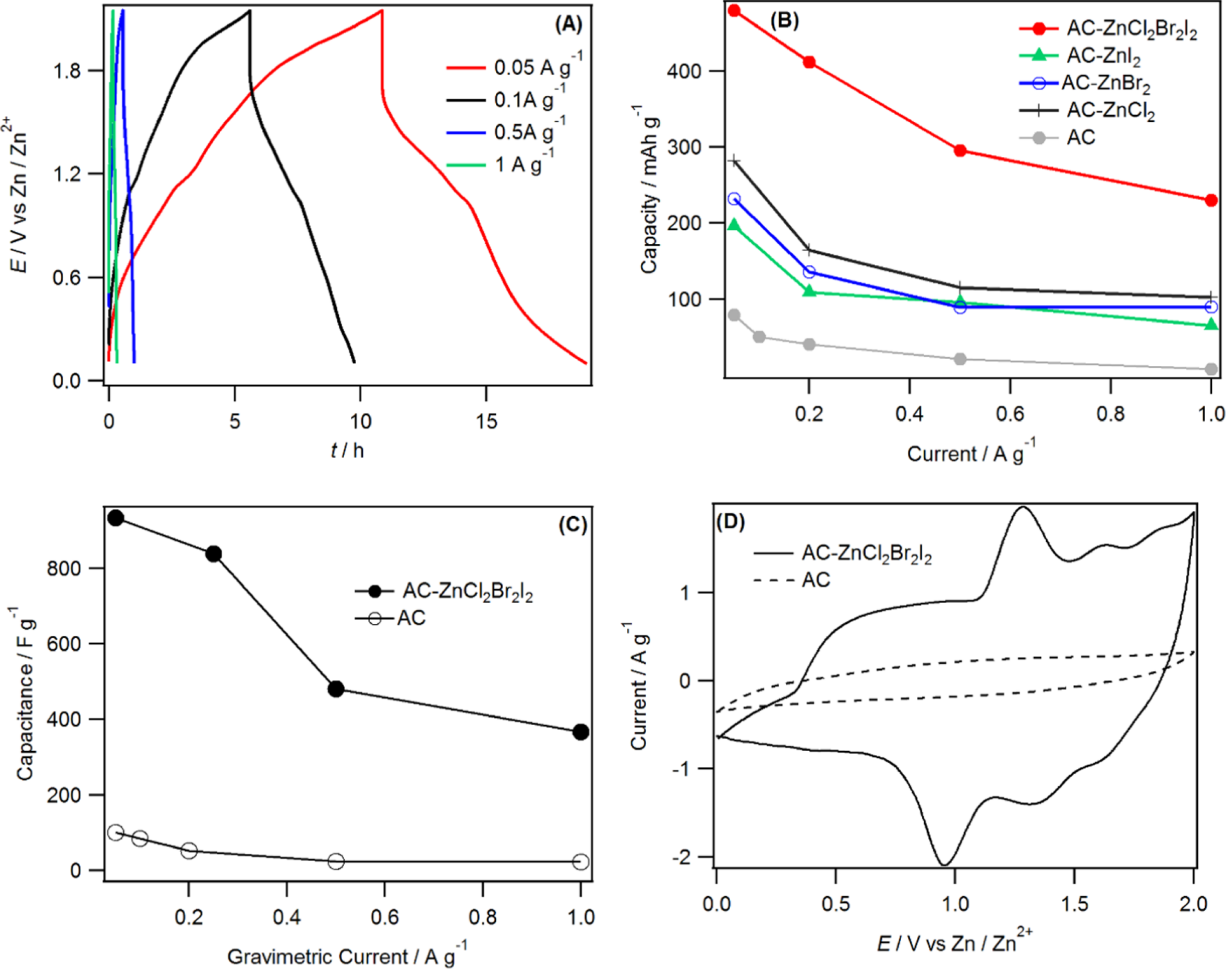
(A) Charge–discharge curve obtained using coin cells constructed from the AC–ZnCl_2_Br_2_I_2_ positive electrode and the Zn foil negative electrode at gravimetric currents indicated. In each case, the voltage range was between 0.1 and 2.15 V vs Zn/Zn^2+^and the gel electrolyte was WiTS. (B) Specific capacity vs gravimetric current using positive electrodes indicated. (C) Gravimetric capacitance vs gravimetric current using AC–ZnCl_2_Br_2_I_2_ and bare AC positive electrodes. (D) CVs recorded at bare AC and AC–ZnCl_2_Br_2_I_2_ cathodes using the Zn anode at 3.0 mV s^–1^ in the WiTS gel electrolyte.

Given that the conversion–adsorption process is the dominant reversible process in Zn halide–carbon composites, the working hypothesis was that variation of the surface area of carbon would determine the extent of cell performance. To this end, various Zn halides were mixed with high surface area-activated carbon (AC) and their performance was tested in full-cell coin cells. Figures 4A and S5 show the representative charge–discharge curves of Zn|AC–ZnX_2_ batteries using the WiTS gel electrolyte at various current densities between 0.1 and 2.0 V. Significantly, the charge–discharge curves possess a near-triangular shape with little deviation from an ideal capacitor response. This implies that the kinetics of the halogen conversion–adsorption reaction at the activated carbon electrode is extremely facile in the WiTS gel electrolyte. However, the CV response at each electrode was not strictly pseudocapacitive due to the presence of redox peaks (see Figures 4D and S6A). The specific capacities for Zn|AC–ZnCl_2_, Zn|AC–ZnBr_2_, and Zn|AC–ZnI_2_ cells are approximately 281, 232, and 196 mAh g^–1^, respectively, at a current density of 0.05 A g^–1^ (compared to <100 mAh g^–1^ for bare AC in the same electrolyte). These capacities decreased to 102, 90, and 65 mAh g^–1^ when the current density was increased to 1.0 A g^–1^, and the corresponding capacity fade is over 60% for each cell (Figure 4B). The capacity loss is most likely due to the low conductivity of the activated carbon and future work will focus on the use of conducting additives, such as carbon black, to further optimize the performance. The capacities obtained at these electrodes are nonetheless higher than Zn-ion capacitors using an activated carbon cathode and Zn anode.^42,43^

The AC composite cathode made from the combination between ZnCl_2_, ZnBr_2_, and ZnI_2_ with the equimolar ratio of the halides achieved specific capacities twice that of individual ZnX_2_ at all current densities studied (Figure 4B). The improved capacity in the Zn|AC–ZnCl_2_Br_2_I_2_ cell relative to pure Zn|ZnX_2_ could be due to a denser adsorption of halide ions on the carbon microstructure. The enhancement in performance of the mixed halide cathode is possibly due to the reduction of Coulombic repulsion of the adsorbed halides and an electrocatalytic effect. It has been shown that the intercalation/adsorption density of halogen on a carbon surface is approximately twice as large as Li–GIC. This is because the oxidation state of the halogen is close to zero, which reduces the average effective charge per halogen atom. This minimizes the Coulombic repulsion and, in turn, increases the adsorption density of halogen on the carbon pore.^6^ Furthermore, the Coulombic repulsion is expected to be further reduced within the carbon microstructure host when the three-halide species (Cl^–^, Br^–^, and I^–^) are adsorbed next to one another. The low Coulombic repulsion in the AC–ZnCl_2_Br_2_I_2_ electrode over individual AC–ZnX_2_ can enhance the packing density of halogens on the carbon surface. Indeed, Yang et al. also observed that the performance of individual LiBr–carbon or LiCl–carbon cathodes is inferior to the binary combination of the LiClBr–carbon cathode.^6^

The halide ion conversion–adsorption reaction is an electrocatalytic process where the reaction is greatly affected by the surface composition of the electrode.^44,45^ CVs were used to see if this electrocatalytic effect is in play, and Figures 4D and S6A show the CVs recorded for each electrode. At the AC–ZnCl_2_Br_2_I_2_, a sharp oxidation peak at 1.18 V due to the conversion and adsorption of iodide species was seen along with a corresponding reduction peak at 1.06 V due to the reduction and recombination of iodide with Zn. Electrochemical data also suggest that the I^–^/I_2_ redox reaction is a surface-controlled process at the AC electrode as both *i*_p,a_ and *i*_p,c_ are proportional to the scan rate (Figure S6C). The Δ*E*_P_ for the I^–^/I_2_ redox couple at AC–ZnCl_2_Br_2_I_2_ was 0.12 V in contrast to over 1.0 V at AC–ZnI_2_. This demonstrates that the I^–^/I_2_ redox reaction is kinetically much faster for the AC–ZnCl_2_Br_2_I_2_ case than the AC–ZnI_2_ system. Similarly, the Δ*E*_P_ for the Br^–^/Br_2_ redox couple decreased from 0.65 V at AC–ZnBr_2_ to 0.1 V at AC–ZnCl_2_Br_2_I_2_, which confirms the electrocatalytic effect of the ternary halide mixture over the individual halides. In other words, the preadsorption of iodine, due to its low redox potential, on the AC surface improved the kinetics of bromine adsorption, which in turn improves the kinetics of chlorine adsorption similar to an electrodeposition process. *Ex situ* XPS was used to gauge the surface composition of fully charged Zn|AC–ZnCl_2_Br_2_I_2_ cell as shown in Figure S7. XPS shows the presence of Cl^–^, Br^–^, and I^–^ for the sample that was analyzed at OCP. However, the XPS signal due to all of the three-halogen species disappeared for the fully charged sample. This indicates that the halide ions are fully oxidized to molecular halogen and coadsorbed to the AC pore, which can sublime under the ultrahigh vacuum of the XPS. Nevertheless, the presence of preadsorbed halogen on the AC surface was confirmed using *in situ* Raman spectroscopy using AC–ZnI_2_ as an example electrode. As shown in Figure S8, the fully charged electrode displayed bands associated with surface-bound I_2_ species as well as the typical defective (*D*) and G-band of AC. The SEM images of the AC–ZnCl_2_B_2_rI_2_ electrode at OCP and after full charging are also presented in Figure S9. The images after charging showed a pore swelling with significant size expansion due to the adsorption of halogens. Each of the expanded pores contained several smaller holes. The small pores could be created by the evaporation of the adsorbed halogen under the SEM-operating vacuum that leaves holes behind.

**Figure 5:**
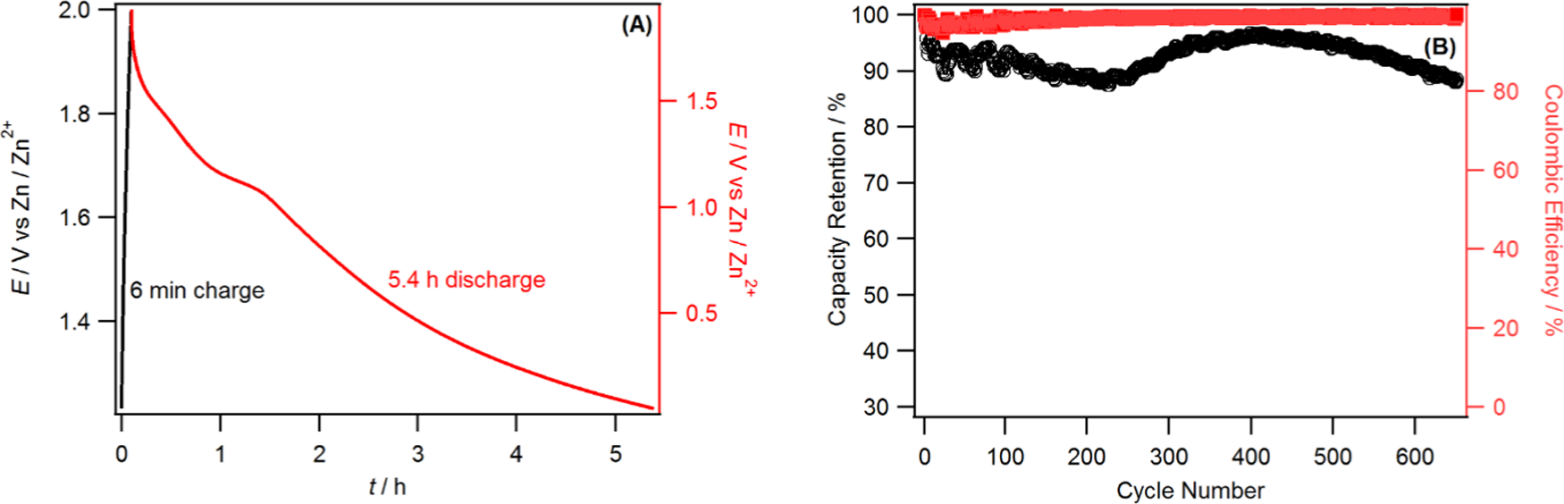
(A) Zn|AC–ZnCl_2_Br_2_I_2_ coin cells charged at 1.0 A g^–1^ (left-hand vertical axis) and discharged at 0.05 A g^–1^ (right-hand vertical axis) using the WiTS gel electrolyte and (B) capacity retention and Coulombic efficiency of the Zn|AC–ZnCl_2_Br_2_I_2_ cell cycled at 0.75 A g^–1^ using the WiTS gel electrolyte.

The specific capacity of Zn|AC–ZnCl_2_Br_2_I_2_ is 479 mAh g^–1^ at 0.05 A g^–1^, which decreased to 230 mAh g^–1^ at 1.0 A g^–1^. The gravimetric capacitances of Zn|AC–ZnCl_2_Br_2_I_2_ ranged from 400 to 930 F g^–1^ depending on the applied current density (Figure 4C). This figure also demonstrates the importance of the Zn halides to the cathode chemistry, proving that the cathodic process involved the immobilized Zn halides. Based on the mass of the cathode (mass of activated carbon plus mass of ZnX_2_), the energy densities of 422 and 160 Wh kg^–1^ at power densities of 122.8 and 1071.7 W kg^–1^ were obtained. These values are higher than the energy density of all other cathode materials reported to date for ZIBs including MnO_2_,^46,47^ V_2_O_5_,^12,48^ Zn_3_V_2_O_7_·2H_2_O,^49^ Zn_0.25_V_2_O_5_·*n*H_2_O,^11^ CuHCF,^50^ and VS_2_ nanosheets.^51^ The cell energy density ranged between 55 Wh kg^–1^ (at 1.0 A g^–1^) and 140 Wh kg^–1^ (at 0.05 A g^–1^), assuming that the weight of the cathode material within a pouch cell configuration is a third of the total mass of the cell.^46^ These values are much higher than those of typical commercial supercapacitors (5–10 Wh kg^–1^), lead-acid batteries (30–40 Wh kg^–1^), and Zn-ion capacitors (17–30 Wh kg^–1^).^52−56^ The energy density of the Zn|AC–ZnCl_2_Br_2_I_2_ cell is even higher than a Li-ion capacitor, where the energy density varies between 30 and 90 Wh kg^–1^.^57^ Li-ion capacitors often use intercalation-type anodes and adsorption-type cathodes (adsorption of large complex anions ([PF_6_]^−^, [TFSI]^−^, [BF_4_]^−^, etc.)), where their overall energy density is limited by the capacitor-type electrode.^57^ The advantages of using halide ion conversion–adsorption within carbon cathodes are (i) they are smaller than most organic anions so that the migration/diffusion of ions is faster, (ii) they are inside the carbon structure so that they do not have to diffuse to the surface from bulk electrolyte, and (iii) they undergo reversible fast redox reactions, which substantially provide an extra charge, unlike inert anions.^6^ The combination of these factors is responsible for the high performance of the Zn|AC–ZnCl_2_Br_2_I_2_ cell. The cell also truly combines the characteristic high energy density of a battery with the high power density of a supercapacitor device. For example, this cell can be fully charged within a few minutes (6 min) at high power and can be discharged for over 5 h at lower rates (see Figure 5A).

The Zn|AC–ZnCl_2_Br_2_I_2_ cell also exhibited excellent cyclic stability when the cell was cycled at 0.75 A g^–1^. The cell capacity retention is 95% after 500 cycles with 99% Coulombic efficiency throughout the cycles (Figure 5B). Although the capacity initially decreased by 10%, a subsequent increase in capacity was observed after 250 cycles and the ohmic drop of the cell decreased with increasing cycling (Figure S10). The increase in capacity could be due to the gradual activation of the electrode, which increases the number of active electrochemical sites for ion adsorption. Finally, the WiTS gel electrolyte performance was tested using a traditional α-MnO_2_ cathode and Figure S11 shows the charge–discharge curve obtained for a Zn|α-MnO_2_ cell. The gel electrolyte exhibited a Coulombic efficiency of over 99% with specific capacities that were increased from 162 to 210 mAh g^–1^ after 50 cycles. This indicates that the WiTS gel electrolyte is as efficient as traditional aqueous electrolytes (such as 1 M ZnSO_4_), but with additional advantages such as the absence of parasitic water reduction reactions, elimination of the need for a separator, and other advantages that semisolid state devices provide, e.g., flexibility. The combination between the WiTS gel electrolyte and ZnX_2_–carbon composite cathode in conjunction with aqueous gel electrolytes offers the tantalizing possibility of solving the issues of poor ZIB performance. This study also provided the very first fundamental understanding of halogen conversion chemistry inside crystalline and amorphous carbon.

## 3 Conclusions

A Zn-conducting water-in-trisalt gel electrolyte and halogen-incorporating cathode have been successfully developed and used in Zn-based electrochemical energy storage for the first time. The benefits of using confined halogen within the carbon structure as a cathode are (i) elimination of the irreversible binding of Zn^2+^ to the host structure within the cathode chemistry, (ii) the provision of substantial extra charge through their conversion–intercalation/adsorption process, and (iii) obviating the need for ions to diffuse to the surface from bulk electrolyte as they are already inside the carbon structure. The most significant findings emerging from this study are that the identity of the Zn halide and carbon structure in the cathode composite produces electrochemical energy storage devices that are fundamentally different from each other (battery vs supercapacitor). The use of graphite in the composite electrode produced batterylike behavior, where the voltage plateau was related to the standard potential of the halogen species. *In situ* Raman spectroelectrochemistry revealed that the identity of halides determines the mechanism of charge storage, intercalation vs electrosorption. In contrast, when activated carbon was used in the composite, the cell acted as a hybrid Zn-ion capacitor due to the fast reversible halogen species electrosorption/desorption in the carbon pores. In this case, the overall capacity is related to the number (binary or ternary) of Zn halides present; and the Zn|(activated carbon–ZnCl_2_Br_2_I_2_) cell exhibited a high specific capacity and energy density as well as good cycling stability. The combination of the Zn|(activated carbon–ZnCl_2_Br_2_I_2_) cell with the WiTS gel electrolyte is promising for the development of high-performing, low-cost, and environmentally friendly energy storage device based on Zn. Future work should focus on the microstructural design of carbon pores to match the size of halogens as well as investigating other carbon structures such as heteroatom-doped carbon or high surface area hard carbon materials.

## 4 Experimental Methods

### 4.1 Materials and Apparatus

All chemicals are of analytical grade and obtained from Sigma-Aldrich, Fluorochem or Alfa Aesar and used as received. X-ray photoelectron spectroscopy (XPS) was performed using a Kratos Axis Ultra DLD spectrometer with a monochromated Al Kα X-ray source (*E* = 1486.6 eV, 10 mA emission). Scanning electron microscope (SEM) analysis was carried out using a FEI Quanta 650 FEG environmental SEM. Powder X-ray diffraction analysis was performed using a Philips X’pert PRO diffractometer with Cu Kα radiation (λ = 0.154 nm) and operating at 40 kV and 30 mA.

### 4.2 WiTS Gel Electrolyte Preparation

Two grams of ZnSO_4_·7H_2_O (99.99%, Sigma-Aldrich), 2 g of zinc trifluoromethanesulfonate (98%, Fluorochem), and 4 g of lithium bis(trifluoromethanesulfonyl)imide (99%, Fluorochem) were mixed together with 1.5 g of ultrapure water (Milli-Q, 18 MΩ cm resistivity) in a mortar and pestle until a uniform white paste formed. Subsequently, 1.3 g (10% of total salt) of a 60% PTFE suspension was added and mixed with a mortar and pestle. The mixture was then heated at 80 °C on a hot plate for 30 min to remove any excess water. A semisolid elastic gel was formed, which can be changed into any desired shape (Figure 1A).

### 4.3 Electrode Preparation

For the preparation of the carbon–ZnX_2_ cathode, first the desired amount of ZnX_2_ (anhydrous ZnCl_2_, ZnBr_2_, ZnI_2_, or mixture of two or more halides) was dissolved in 0.5 g of water followed by slow addition of natural graphite (−325 mesh, 99.8%, Sigma-Aldrich) or activated carbon (YEC-8B, Fuzhou Yihuan Carbon Co., Ltd) while mixing homogeneously with a mortar and pestle. The mass ratio between ZnX_2_ and carbon was 1:3. PTFE suspension (5% with respect to the mass of carbon) was added to the thick slurry and mixed to uniformly coat the mixture with the polymer binder. The excess water was removed by heating the mixture on a hot plate. The resulting carbon–ZnX_2_ clay was quite flexible and could be made as a free-standing film or rolled onto a prepunched (15 mm diameter) titanium (99.99%, Alfa Aeser) foil current collector. The composite electrode was dried in a vacuum oven at 80 °C overnight. The typical mass loading of the carbon–ZnX_2_ composite electrode ranged from 2 to 5 mg cm^–2^.

### 4.4 Battery Assembly and Electrochemical Measurements

The full cells were assembled in CR2032-type coin cells using carbon–ZnX_2_ as the cathode and Zn foil as the anode. The flexible gel electrolyte was spread onto the Zn foil with an approximate thickness of 0.5–1.0 mm and acted as both the electrolyte and separator. The coin cell was sealed using a hydraulic crimping machine (MSK-160D) in an ambient atmosphere. Three-electrode cell electrochemical measurements were conducted using a WiTS gel electrolyte that was rolled onto a microscopy glass slide for electrode connection. A glassy carbon working electrode, Pt wire counter electrode, and Zn metal reference electrode were used. Electrochemical measurements were performed using an Autolab potentiostat (model PGSTAT302N, Metrohm Autolab, The Netherlands). The charge–discharge battery tests were carried out using a Basytec Cell Test System (BaSyTecGmbH, Asselfingen, Germany) with 32 independent test channels. The average capacity of three different coin cells was used to report capacity/capacitance.

### 4.5 *In Situ* Raman Spectroscopy Measurement

Raman spectra were obtained using a Renishaw inVia microscope with a 532 nm excitation laser operated at a power of 0.274 mW with a grating of 1800 lines/mm and 50× objective. The *in situ* Raman cell was obtained from ECC-Opto-Std (EL-Cell GmbH, Hamburg, Germany) and the cell was comprised of a free-standing carbon–ZnX_2_ positive electrode and a Zn foil negative electrode with a WiTS gel electrolyte. A titanium foil that contained a small hole in its middle (diameter ca. 1 mm) was used as a current collector for the positive electrode. The exciting laser beam was shone through a thin glass window onto the rear of the free-standing carbon–ZnX_2_ film through the small hole in the center of the Ti foil. Spectral scans were collected in a backscattering configuration. The Raman measurements were collected at various voltages as the cell charged and discharged at 1 mV s^–1^.

## Supporting Information Available

Cyclic voltammograms and galvanostatic charge–discharge curves of various electrodes, XPS, Raman spectroscopy and SEM of charged electrodes, and AC electrochemical impedance spectroscopy of water-in-salt gel electrolyte (PDF)

## References

[ref1] Pan H. L.; Shao Y. Y.; Yan P. F.; Cheng Y. W.; Han K. S.; Nie Z. M.; Wang C. M.; Yang J. H.; Li X. L.; Bhattacharya P.; Mueller K. T.; Liu J. Reversible Aqueous Zinc/Manganese Oxide Energy Storage from Conversion Reactions. Nat. Energy 2016, 1, 16039 10.1038/nenergy.2016.39.

[ref2] Song M.; Tan H.; Chao D. L.; Fan H. J. Recent Advances in Zn-Ion Batteries. Adv. Funct. Mater. 2018, 28, 1802564 10.1002/adfm.201802564.

[ref3] Luo J. Y.; Cui W. J.; He P.; Xia Y. Y. Raising the Cycling Stability of Aqueous Lithium-Ion Batteries by Eliminating Oxygen in the Electrolyte. Nat. Chem 2010, 2, 760–765. 10.1038/nchem.763. 20729897

[ref4] Yesibolati N.; Umirov N.; Koishybay A.; Omarova M.; Kurmanbayeva I.; Zhang Y. G.; Zhao Y.; Bakenov Z. High Performance Zn/Lifepo4 Aqueous Rechargeable Battery for Large Scale Applications. Electrochim. Acta 2015, 152, 505–511. 10.1016/j.electacta.2014.11.168.

[ref5] Suo L. M.; Borodin O.; Gao T.; Olguin M.; Ho J.; Fan X. L.; Luo C.; Wang C. S.; Xu K. ″Water-in-Salt″ Electrolyte Enables High-Voltage Aqueous Lithium-Ion Chemistries. Science 2015, 350, 938–943. 10.1126/science.aab1595. 26586759

[ref6] Yang C. Y.; Chen J.; Ji X.; Pollard T. P.; Lu X. J.; Sun C. J.; Hou S.; Liu Q.; Liu C. M.; Qing T. T.; Wang Y. Q.; Borodin O.; Ren Y.; Xu K.; Wang C. S. Aqueous Li-Ion Battery Enabled by Halogen Conversion-Intercalation Chemistry in Graphite. Nature 2019, 569, 245–250. 10.1038/s41586-019-1175-6. 31068723

[ref7] Wang F.; Borodin O.; Gao T.; Fan X. L.; Sun W.; Han F. D.; Faraone A.; Dura J. A.; Xu K.; Wang C. S. Highly Reversible Zinc Metal Anode for Aqueous Batteries. Nat. Mater. 2018, 17, 543–549. 10.1038/s41563-018-0063-z. 29662160

[ref8] Parker J. F.; Chervin C. N.; Nelson E. S.; Rolison D. R.; Long J. W. Wiring Zinc in Three Dimensions Re-Writes Battery Performance-Dendrite-Free Cycling. Energy Environ. Sci. 2014, 7, 1117–1124. 10.1039/C3EE43754J.

[ref9] Deng Y. P.; Liang R. L.; Jiang G. P.; Jiang Y.; Yu A. P.; Chen Z. W. The Current State of Aqueous Zn-Based Rechargeable Batteries. ACS Energy Lett 2020, 5, 1665–1675. 10.1021/acsenergylett.0c00502.

[ref10] Chen Z.; Tang Y.; Du X. F.; Chen B. B.; Lu G. L.; Han X. Q.; Zhang Y. J.; Yang W. H.; Han P. X.; Zhao J. W.; Cui G. L. Anion Solvation Reconfiguration Enables High-Voltage Carbonate Electrolytes for Stable Zn/Graphite Cells. Angew. Chem., Int. Ed. 2020, 59, 21769–21777. 10.1002/anie.202010423. 32812326

[ref11] Kundu D.; Adams B. D.; Duffort V.; Vajargah S. H.; Nazar L. F. A High-Capacity and Long-Life Aqueous Rechargeable Zinc Battery Using a Metal Oxide Intercalation Cathode. Nat. Energy 2016, 1, 16119 10.1038/nenergy.2016.119.

[ref12] Wang L. L.; Huang K. W.; Chen J. T.; Zheng J. R. Ultralong Cycle Stability of Aqueous Zinc-Ion Batteries with Zinc Vanadium Oxide Cathodes. Sci. Adv. 2019, 5, eaax4279 10.1126/sciadv.aax4279. 32047853 PMC6984968

[ref13] Zhang L. Y.; Chen L.; Zhou X. F.; Liu Z. P. Towards High-Voltage Aqueous Metal-Ion Batteries Beyond 1.5 V: The Zinc/Zinc Hexacyanoferrate System. Adv. Energy Mater. 2015, 5, 1400930 10.1002/aenm.201400930.

[ref14] Gupta T.; Kim A.; Phadke S.; Biswas S.; Luong T.; Hertzberg B. J.; Chamoun M.; Evans-Lutterodt K.; Steingart D. A. Improving the Cycle Life of a High-Rate, High-Potential Aqueous Dual Ion Battery Using Hyper-Dendritic Zinc and Copper Hexacyanoferrate. J. Power Sources 2016, 305, 22–29. 10.1016/j.jpowsour.2015.11.065.

[ref15] Yuan C. L.; Zhang Y.; Pan Y.; Liu X. W.; Wang G. L.; Cao D. X. Investigation of the Intercalation of Polyvalent Cations (Mg2+, Zn2+) into Lambda-Mno2 for Rechargeable Aqueous Battery. Electrochim. Acta 2014, 116, 404–412. 10.1016/j.electacta.2013.11.090.

[ref16] Lee B.; Lee H. R.; Kim H.; Chung K. Y.; Cho B. W.; Oh S. H. Elucidating the Intercalation Mechanism of Zinc Ions into Alpha-Mno2 for Rechargeable Zinc Batteries. Chem. Commun. 2015, 51, 9265–9268. 10.1039/C5CC02585K. 25920416

[ref17] Xu C. J.; Li B. H.; Du H. D.; Kang F. Y. Energetic Zinc Ion Chemistry: The Rechargeable Zinc Ion Battery. Angew. Chem., Int. Ed. 2012, 51, 933–935. 10.1002/anie.201106307. 22170816

[ref18] Alfaruqi M. H.; Mathew V.; Gim J.; Kim S.; Song J.; Baboo J. P.; Choi S. H.; Kim J. Electrochemically Induced Structural Transformation in a Gamma-Mno2 Cathode of a High Capacity Zinc-Ion Battery System. Chem. Mater. 2015, 27, 3609–3620. 10.1021/cm504717p.

[ref19] Lai Q. Z.; Zhang H. M.; Li X. F.; Zhang L. Q.; Cheng Y. H. A Novel Single Flow Zinc-Bromine Battery with Improved Energy Density. J. Power Sources 2013, 235, 1–4. 10.1016/j.jpowsour.2013.01.193.

[ref20] Atkins P. W.; Paula J. D. Physical Chemistry, 9th ed.; Oxford University Press: New York, 2010.

[ref21] Nguyen-Trung C.; Bryan J. C.; Palmer D. A. Crystal Structure and Thermogravimetric Analysis of Hexaaquazinc Triflate. Struct. Chem. 2004, 15, 89–94. 10.1023/B:STUC.0000011243.10406.8b.

[ref22] Moezzi A.; Cortie M. B.; McDonagh A. M. Zinc Hydroxide Sulphate and Its Transformation to Crystalline Zinc Oxide. Dalton Trans. 2013, 42, 14432–14437. 10.1039/c3dt51638e. 23963063

[ref23] Baek B.; Lee S.; Jung C. Pyrrolidinium Cation-Based Ionic Liquids with Different Functional Groups: Butyl, Butyronitrile, Pentenyl, and Methyl Butyrate. Int. J. Electrochem. Sci. 2011, 6, 6220–6234.

[ref24] Guo X.; Zhang Z.; Li J.; Luo N.; Chai G.-L.; Miller T. S.; Lai F.; Shearing P.; Brett D. J. L.; Han D.; Weng Z.; He G.; Parkin I. P. Alleviation of Dendrite Formation on Zinc Anodes Via Electrolyte Additives. ACS Energy Lett. 2021, 395–403. 10.1021/acsenergylett.0c02371.

[ref25] Zhao Z.; Zhao J.; Hu Z.; Li J.; Li J.; Zhang Y.; Wang C.; Cui G. Long-Life and Deeply Rechargeable Aqueous Zn Anodes Enabled by a Multifunctional Brightener-Inspired Interphase. Energy Environ. Sci. 2019, 12, 1938–1949. 10.1039/C9EE00596J.

[ref26] Qiu H. Y.; Du X. F.; Zhao J. W.; Wang Y. T.; Ju J. W.; Chen Z.; Hu Z. L.; Yan D. P.; Zhou X. H.; Cui G. L. Zinc Anode-Compatible in situ Solid Electrolyte Interphase Via Cation Solvation Modulation. Nat. Commun. 2019, 10, 5374 10.1038/s41467-019-13436-3. 31772177 PMC6879498

[ref27] Xu M.; Ivey D. G.; Qu W.; Xie Z.; Dy E.; Yuan X. Z. Zn/Zn(Ii) Redox Kinetics and Zn Deposit Morphology in Water Added Ionic Liquids with Bis(Trifluoromethanesulfonyl)Imide Anions. J. Electrochem. Soc. 2014, 161, A128–A136. 10.1149/2.058401jes.

[ref28] Zhang Q.; Luan J. Y.; Fu L.; Wu S. G.; Tang Y. G.; Ji X. B.; Wang H. Y. The Three-Dimensional Dendrite-Free Zinc Anode on a Copper Mesh with a Zinc-Oriented Polyacrylamide Electrolyte Additive. Angew. Chem., Int. Ed. 2019, 58, 15841–15847. 10.1002/anie.201907830. 31437348

[ref29] Gaier J. R.; Ditmars N. F.; Dillon A. R. Aqueous Electrochemical Intercalation of Bromine into Graphite Fibers. Carbon 2005, 43, 189–193. 10.1016/j.carbon.2004.09.005.

[ref30] Axdal S. H. A.; Chung D. D. L. Kinetics and Thermodynamics of Intercalation of Bromine in Graphite. 1. Experimental. Carbon 1987, 25, 191–210. 10.1016/0008-6223(87)90117-5.

[ref31] Lowe E. R.; Banks C. E.; Compton R. G. Edge Plane Pyrolytic Graphite Electrodes for Halide Detection in Aqueous Solutions. Electroanalysis 2005, 17, 1627–1634. 10.1002/elan.200503267.

[ref32] Behrens P.; Beuthien H.; Eickhoff H. P.; Metz W.; Niemann W. Structural Investigations of the Graphite-Intercalation Compounds of the Dichlorides of the Ii_b_-Elements (Zn. Cd, Hg). Synth. Met. 1988, 23, 95–100. 10.1016/0379-6779(88)90467-5.

[ref33] Beltran J. J.; Barrero C. A.; Punnoose A. Relationship between Ferromagnetism and Formation of Complex Carbon Bonds in Carbon Doped Zno Powders. Phys. Chem. Chem. Phys. 2019, 21, 8808–8819. 10.1039/C9CP01277J. 30968907

[ref34] Rubim J. C.; Sala O. Raman-Spectra of Chemisorbed Bromine and Iodine on Zeolites. J. Raman Spectrosc. 1980, 9, 155–156. 10.1002/jrs.1250090305.

[ref35] Jung N.; Crowther A. C.; Kim N.; Kim P.; Brus L. Raman Enhancement on Graphene: Adsorbed and Intercalated Molecular Species. ACS Nano 2010, 4, 7005–7013. 10.1021/nn102227u. 20945922

[ref36] Hung C. C.; Kucera D. Graphite-Intercalation Compound with Iodine as the Major Intercalate.. Carbon 1994, 32, 1441–1448. 10.1016/0008-6223(94)90138-4.

[ref37] Lu K.; Hu Z. Y.; Ma J. Z.; Ma H. Y.; Dai L. M.; Zhang J. T. A Rechargeable Iodine-Carbon Battery That Exploits Ion Intercalation and Iodine Redox Chemistry. Nat. Commun. 2017, 8, 527 10.1038/s41467-017-00649-7. 28904375 PMC5597605

[ref38] Erbil A.; Dresselhaus G.; Dresselhaus M. S. Raman-Scattering as a Probe of Structural Phase-Transitions in the Intercalated Graphite-Bromine System. Phys. Rev. B 1982, 25, 5451–5460. 10.1103/PhysRevB.25.5451.

[ref39] Ejigu A.; Le Fevre L. W.; Fujisawa K.; Terrones M.; Forsyth A. J.; Dryfe R. A. W. Electrochemically Exfoliated Graphene Electrode for High-Performance Rechargeable Chloroaluminate and Dual-Ion Batteries. ACS Appl. Mater. Interfaces 2019, 11, 23261–23270. 10.1021/acsami.9b06528. 31252480

[ref40] Ding J.; Hu W. B.; Paek E.; Mitlin D. Review of Hybrid Ion Capacitors: From Aqueous to Lithium to Sodium. Chem. Rev. 2018, 118, 6457–6498. 10.1021/acs.chemrev.8b00116. 29953230

[ref41] Stammreich H.; Forneris R.; Tavares Y. High-Resolution Raman Spectroscopy in the Red and near Infrared. 2. Vibrational Frequencies and Molecular Interactions of Halogens and Diatomic Interhalogens. Spectrochim. Acta 1961, 17, 1173–1184. 10.1016/0371-1951(61)80165-3.

[ref42] Dong L. B.; Ma X. P.; Li Y.; Zhao L.; Liu W. B.; Cheng J. Y.; Xu C. J.; Li B. H.; Yang Q. H.; Kang F. Y. Extremely Safe, High-Rate and Ultralong-Life Zinc-Ion Hybrid Supercapacitors. Energy Storage Mater 2018, 13, 96–102. 10.1016/j.ensm.2018.01.003.

[ref43] Chen S. M.; Ma L. T.; Zhang K.; Kamruzzaman M.; Zhi C. Y.; Zapien J. A. A Flexible Solid-State Zinc Ion Hybrid Supercapacitor Based on Co-Polymer Derived Hollow Carbon Spheres. J. Mater. Chem. A. 2019, 7, 7784–7790. 10.1039/C9TA00733D.

[ref44] Liu A. H.; Xu L. A.; Li T.; Dong S. J.; Wang E. K. Electrocatalytic Oxidation and Ion-Chromatographic Detection of Br-, I-, So32-, S2o32- and Scn- at a Platinum Particle-Based Glassy-Carbon Modified Electrode. J. Chromatogr. A 1995, 699, 39–47. 10.1016/0021-9673(95)00032-I.

[ref45] Wang M. K.; Anghel A. M.; Marsan B.; Ha N. L. C.; Pootrakulchote N.; Zakeeruddin S. M.; Grätzel M. Cos Supersedes Pt as Efficient Electrocatalyst for Triiodide Reduction in Dye-Sensitized Solar Cells. J. Am. Chem. Soc. 2009, 131, 15976–15977. 10.1021/ja905970y. 19845335

[ref46] Zhang N.; Cheng F. Y.; Liu Y. C.; Zhao Q.; Lei K. X.; Chen C. C.; Liu X. S.; Chen J. Cation-Deficient Spinel Znmn2o4 Cathode in Zn(Cf3so3)(2) Electrolyte for Rechargeable Aqueous Zn-Ion Battery. J. Am. Chem. Soc. 2016, 138, 12894–12901. 10.1021/jacs.6b05958. 27627103

[ref47] Zhang N.; Cheng F. Y.; Liu J. X.; Wang L. B.; Long X. H.; Liu X. S.; Li F. J.; Chen J. Rechargeable Aqueous Zinc-Manganese Dioxide Batteries with High Energy and Power Densities. Nat. Commun. 2017, 8, 405 10.1038/s41467-017-00467-x. 28864823 PMC5581336

[ref48] Yan M. Y.; He P.; Chen Y.; Wang S. Y.; Wei Q. L.; Zhao K. N.; Xu X.; An Q. Y.; Shuang Y.; Shao Y. Y.; Mueller K. T.; Mai L. Q.; Liu J.; Yang J. H. Water-Lubricated Intercalation in V2o5 Center Dot Nh(2)O for High-Capacity and High-Rate Aqueous Rechargeable Zinc Batteries.. Adv. Mater. 2018, 30, 1703725 10.1002/adma.201703725. 29131432

[ref49] Xia C.; Guo J.; Lei Y. J.; Liang H. F.; Zhao C.; Alshareef H. N. Rechargeable Aqueous Zinc-Ion Battery Based on Porous Framework Zinc Pyrovanadate Intercalation Cathode. Adv. Mater. 2018, 30, 1705580 10.1002/adma.201705580. 29226488

[ref50] Trócoli R.; La Mantia F. An Aqueous Zinc-Ion Battery Based on Copper Hexacyanoferrate. ChemSusChem 2015, 8, 481–485. 10.1002/cssc.201403143. 25510850

[ref51] He P.; Yan M. Y.; Zhang G. B.; Sun R. M.; Chen L. N.; An Q. Y.; Mai L. Q. Layered Vs2 Nanosheet-Based Aqueous Zn Ion Battery Cathode. Adv. Energy Mater. 2017, 7, 1601920 10.1002/aenm.201601920.

[ref52] Huang Y.; Liu J.; Huang Q.; Zheng Z.; Hiralal P.; Zheng F.; Ozgit D.; Su S.; Chen S.; Tan P.-H.; Zhang S.; Zhou H. Flexible High Energy Density Zinc-Ion Batteries Enabled by Binder-Free Mno2/Reduced Graphene Oxide Electrode. npj Flexible Electron. 2018, 2, 21 10.1038/s41528-018-0034-0.

[ref53] Wu S. L.; Chen Y. T.; Jiao T. P.; Zhou J.; Cheng J. Y.; Liu B.; Yang S. R.; Zhang K. L.; Zhang W. J. An Aqueous Zn-Ion Hybrid Supercapacitor with High Energy Density and Ultrastability up to 80 000 Cycles. Adv. Energy Mater. 2019, 9, 1902915 10.1002/aenm.201902915.

[ref54] Zhang H. Z.; Liu Q. Y.; Fang Y. B.; Teng C. L.; Liu X. Q.; Fang P. P.; Tong Y. X.; Lu X. H. Boosting Zn-Ion Energy Storage Capability of Hierarchically Porous Carbon by Promoting Chemical Adsorption. Adv. Mater. 2019, 31, 1904948 10.1002/adma.201904948. 31523863

[ref55] He L.; Liu Y.; Li C. Y.; Yang D. Z.; Wang W. G.; Yan W. Q.; Zhou W. B.; Wu Z. X.; Wang L. L.; Huang Q. H.; Zhu Y. S.; Chen Y. H.; Fu L. J.; Hou X. H.; Wu Y. P. A Low-Cost Zn-Based Aqueous Supercapacitor with High Energy Density. ACS Appl. Energy Mater. 2019, 2, 5835–5842. 10.1021/acsaem.9b00981.

[ref56] Wang H.; Wang M.; Tang Y. B. A Novel Zinc-Ion Hybrid Supercapacitor for Long-Life and Low-Cost Energy Storage Applications. Energy Storage Mater. 2018, 13, 1–7. 10.1016/j.ensm.2017.12.022.

[ref57] Wang X. J.; Liu L. L.; Niu Z. Q. Carbon-Based Materials for Lithium-Ion Capacitors. Mater. Chem. Front. 2019, 3, 1265–1279. 10.1039/C9QM00062C.

