# Supplementary material for: Reversible Electrochemical Energy Storage Based on Zinc-Halide Chemistry
Source: ACS Appl Mater Interfaces. 2021 Mar 16;13(12):14112–21. doi: 10.1021/acsami.0c20622 (PMC8041251; doi:10.1021/acsami.0c20622)
Supplement: Supplementary file 1 — am0c20622_si_001.pdf [file am0c20622_si_001.pdf]

Supplementary Information

Reversible Electrochemical Energy Storage based on Zinc-Halide Chemistry

Andinet Ejigu*[a,b], Lewis W. Le Fevre[b,c], and Robert A.W. Dryfe*[a,b, d]

[a]Dept. of Chemistry, University of Manchester, Oxford Road, Manchester M13 9PL, UK

[b]National Graphene Institute, University of Manchester, Oxford Road, Manchester, M13 9PL, UK

[c] Dept. of Electrical and Electronic Engineering, University of Manchester, Oxford Road, Manchester M13 9PL, UK

[d] Henry Royce Institute, University of Manchester, Oxford Road, Manchester, M13 9PL, UK

Corresponding Authors

* Email: robert.dryfe@manchester.ac.uk, Tel: +44 (0)161-306-4522. Fax: +44 (0)161-275-4598.

* Email: andinet.aynalem@manchester.ac.uk

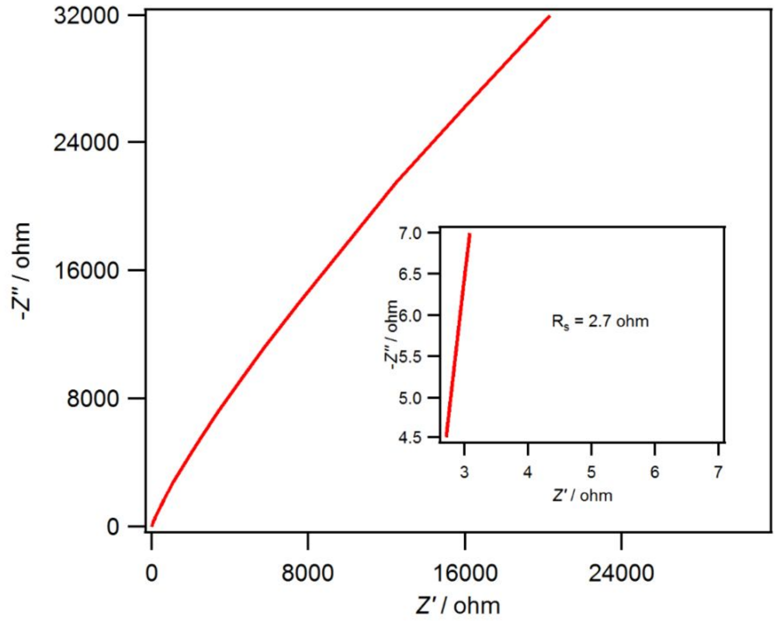

Figure S1 *Electrochemical impedance spectroscopy data obtained using WiTS gel electrolyte sandwiched between two stainless steel circular plates. The thickness of the gel electrolyte was 220 μm. The inset shows the magnified serial resistance*

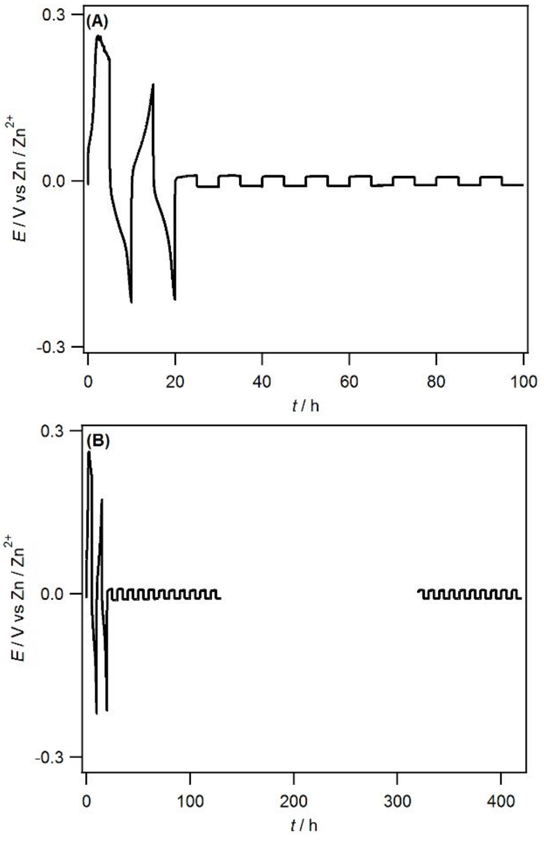

Figure S2 (A) and (B) *Galvanostatic charge-discharge curve obtained using symmetrical Zn* |*Zn cell in WiTS gel electrolyte at 0.1 mA cm^{-2}*

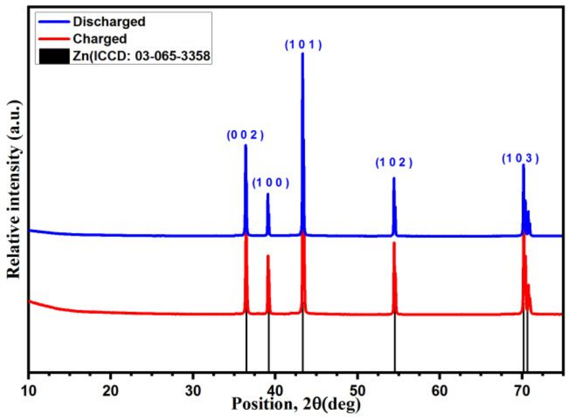

Figure S3 *XRD pattern of a Zn anode and cathode after 40 h stripping/plating process in the WiTS gel electrolyte in a symmetrical Zn/Zn cell.*

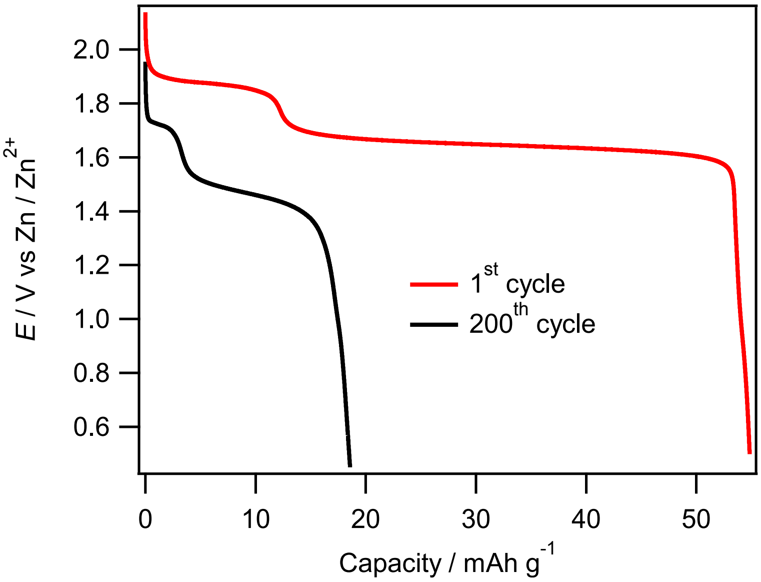

FigureS4. *Galvanostatic discharge curves vs capacity obtained at a current density of 0.05 A* g^{-1} *using (G-*$ZnCl_2$*-*$ZnBr_2$*) positive electrodes and Zn negative electrode in WiTS gel electrolyte.*

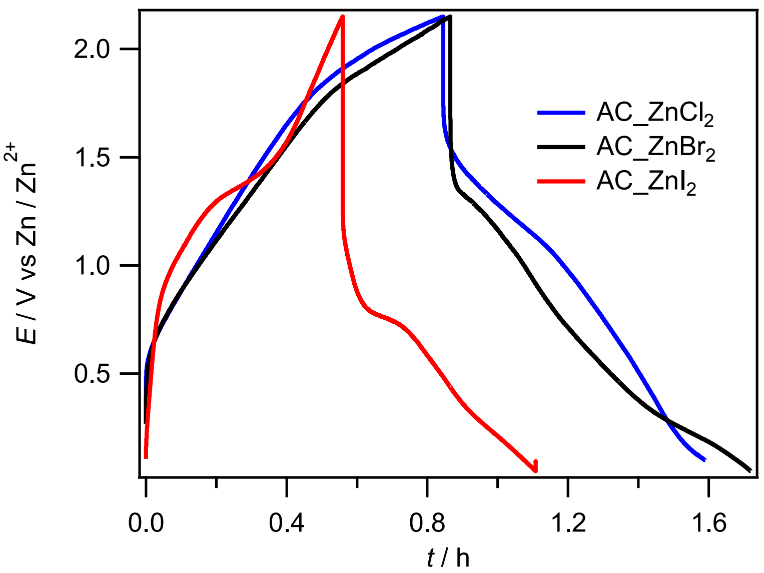

Figure S5. *Charge–discharge curve obtained using coin cells constructed from shown positive electrode and Zn foil negative electrode at 0.2 A g*$^{-1}$ for AC-$ZnCl_2$ & AC-$ZnBr_2$, and at 0.5 *A g*$^{-1}$ for AC-ZnI_2*and using WiTS gel electrolyte*

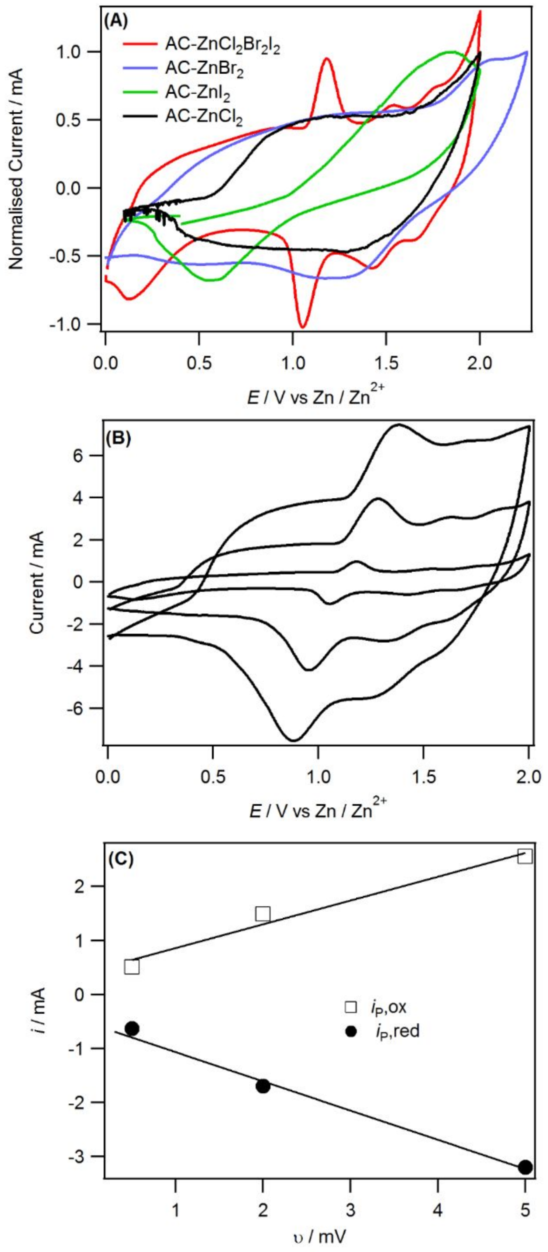

Figure S6 *(A) CVs recorded using various Zn/AC-ZnX_2 coin cells at 1.0 mV s^{-1} in WiTS gel electrolyte. (B) CVs recorded at Zn |AC-Zn$Cl_2Br_2I_2$ coin cells at 0.5, 2, and 3.0 mV s^{-1} (bottom to up) in WiTS gel electrolyte and (C)graphs of peak current versus scan rate for the oxidation and reduction process shown in (A)*

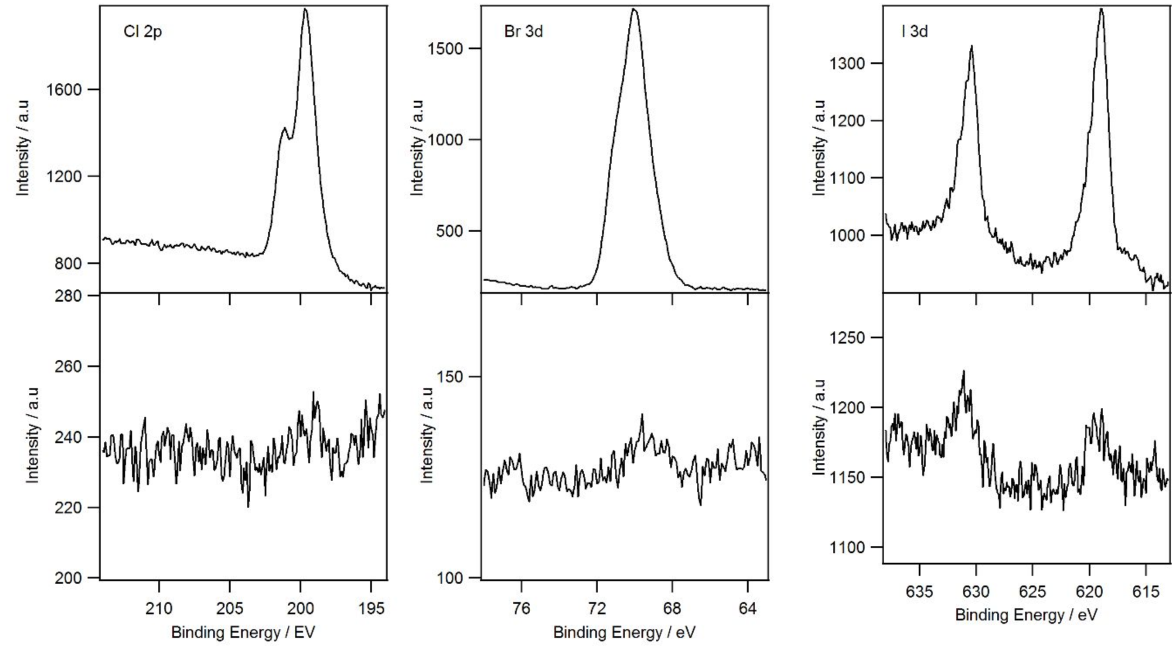

Figure S7 *XPS of AC-ZnCl$_2$Br$_2$I$_2$ (Cl 2p, Br 3d and I 3d) obtained at OCP (for the upper row) and after fully charged (bottom row)*

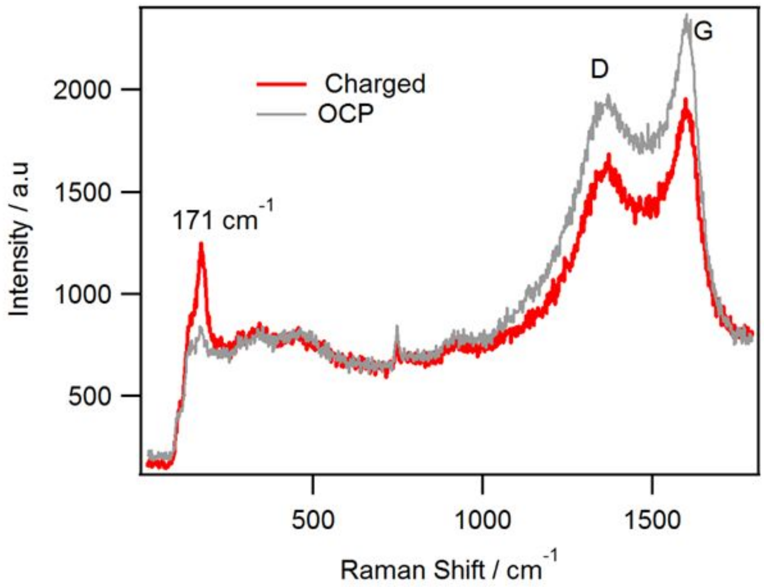

Figure S8 *In situ Raman spectral series of Zn/G-ZnI$_2$ cell in WiTS gel electrolyte at OCP and full charge*

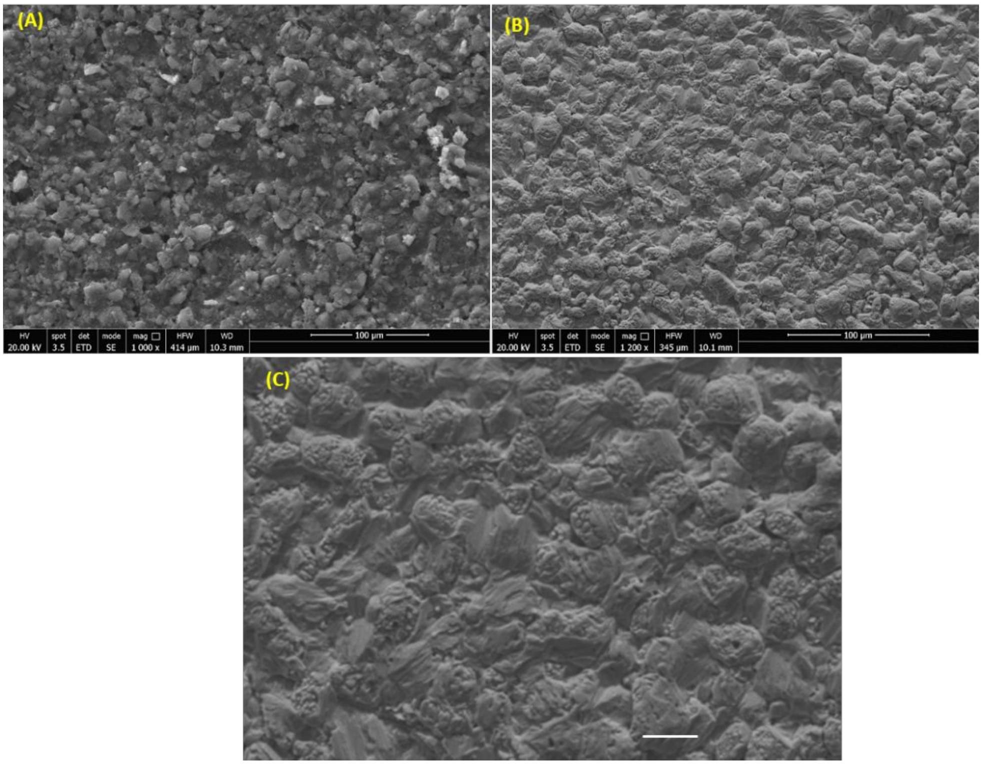

Figure S9 *SEM images of AC-$ZnCl_2Br_2I_2$ electrode (A) at OCP, and (B) &(C) after fully charging. The scale bar in (C) is 10 μm.*

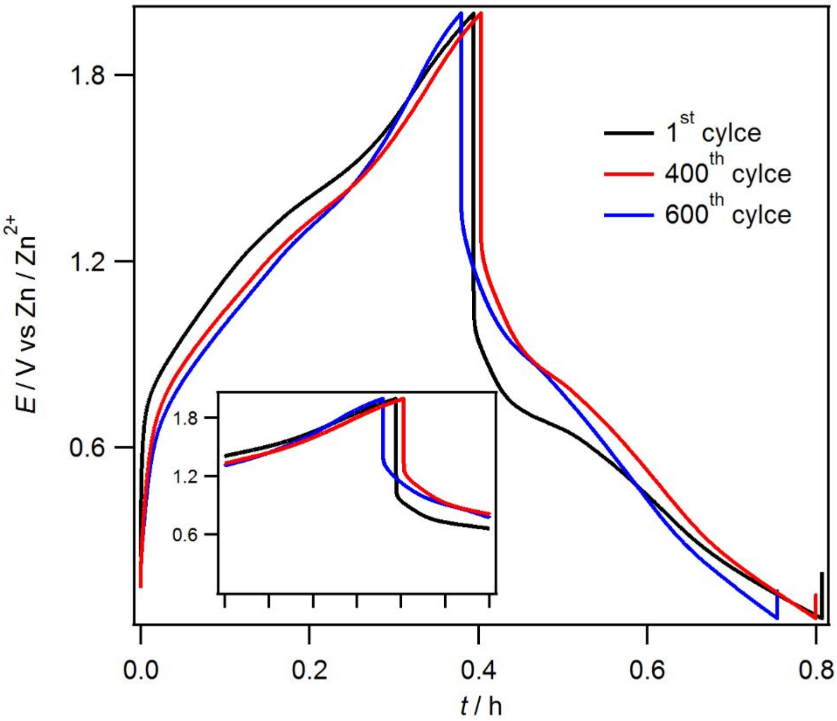

Figure S10. *Charge–discharge curve obtained using Zn/*AC-$ZnCl_2Br_2I_2$ cell *at 0.75 A* g^{-1} *using WiTS gel electrolyte at shown cycling stage.*

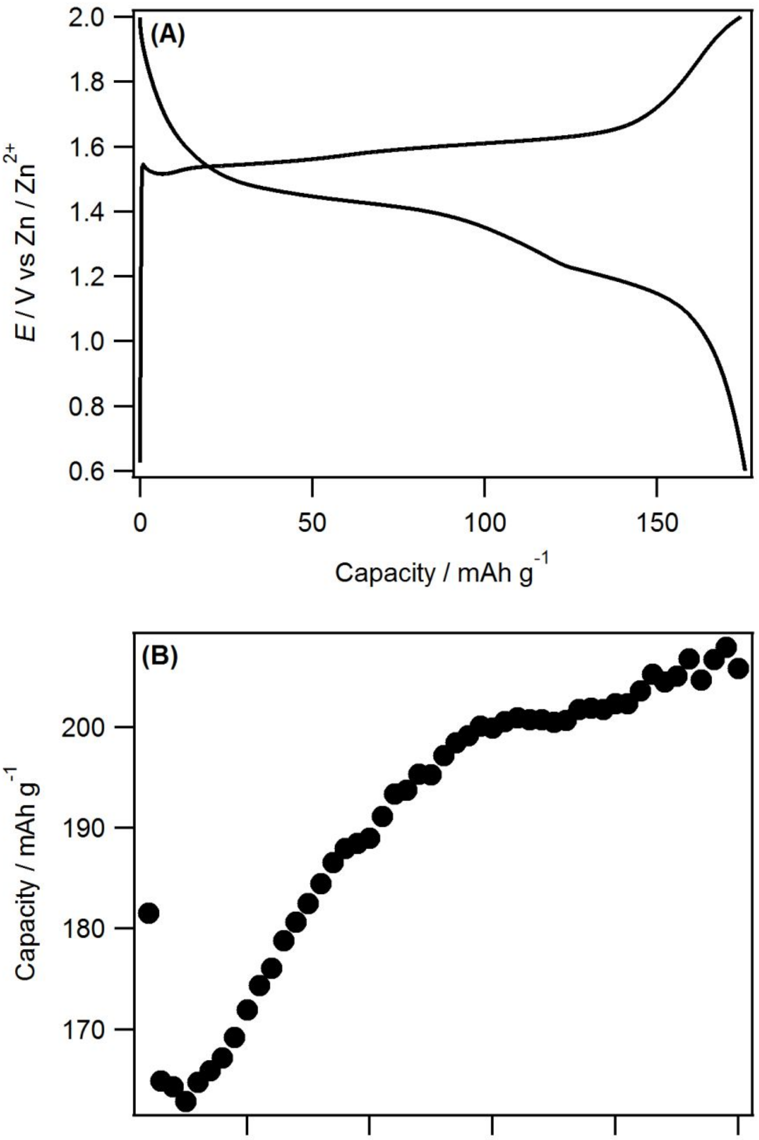

Figure S11 *(A) Galvanostatic discharge curves vs capacity obtained at a gravimetric current density of 0.02A* g^{-1} *using* α*-*MnO_2 *positive electrode and Zn negative electrode in WiTS gel electrolyte and (B) Capacity as a function of cell (Zn/*α*-*MnO_2*) cycling at 0.02 A* g^{-1} *using WiTS gel electrolyte*
